# Local politico-administrative perspectives on quality improvement based on national registry data in Sweden: a qualitative study using the Consolidated Framework for Implementation Research

**DOI:** 10.1186/s13012-014-0189-6

**Published:** 2014-12-28

**Authors:** Mio Fredriksson, Ann Catrine Eldh, Sofie Vengberg, Tobias Dahlström, Christina Halford, Lars Wallin, Ulrika Winblad

**Affiliations:** Department of Public Health and Caring Sciences, Uppsala University, Uppsala, 751 22 Sweden; School of Health and Social Science, Dalarna University, Falun, 791 88 Sweden; Division of Nursing, Department of Neurobiology, Care Sciences and Society, Karolinska Institutet, Huddinge, 141 83 Sweden

**Keywords:** Quality registry, Clinical database, Clinical registry, Implementation, Quality improvement, Consolidated Framework for Implementation Research

## Abstract

**Background:**

Through a national policy agreement, over 167 million Euros will be invested in the Swedish National Quality Registries (NQRs) between 2012 and 2016. One of the policy agreement’s intentions is to increase the use of NQR data for quality improvement (QI). However, the evidence is fragmented as to how the use of medical registries and the like lead to quality improvement, and little is known about non-clinical use. The aim was therefore to investigate the perspectives of Swedish politicians and administrators on quality improvement based on national registry data.

**Methods:**

Politicians and administrators from four county councils were interviewed. A qualitative content analysis guided by the Consolidated Framework for Implementation Research (CFIR) was performed.

**Results:**

The politicians’ and administrators’ perspectives on the use of NQR data for quality improvement were mainly assigned to three of the five CFIR domains. In the domain of *intervention characteristics*, data reliability and access in reasonable time were not considered entirely satisfactory, making it difficult for the politico-administrative leaderships to initiate, monitor, and support timely QI efforts. Still, politicians and administrators trusted the idea of using the NQRs as a base for quality improvement. In the domain of *inner setting*, the organizational structures were not sufficiently developed to utilize the advantages of the NQRs, and readiness for implementation appeared to be inadequate for two reasons. Firstly, the resources for data analysis and quality improvement were not considered sufficient at politico-administrative or clinical level. Secondly, deficiencies in leadership engagement at multiple levels were described and there was a lack of consensus on the politicians’ role and level of involvement. Regarding the domain of *outer setting*, there was a lack of communication and cooperation between the county councils and the national NQR organizations.

**Conclusions:**

The Swedish experiences show that a government-supported national system of well-funded, well-managed, and reputable national quality registries needs favorable local politico-administrative conditions to be used for quality improvement; such conditions are not yet in place according to local politicians and administrators.

**Electronic supplementary material:**

The online version of this article (doi:10.1186/s13012-014-0189-6) contains supplementary material, which is available to authorized users.

## Background

The present study combines policy implementation research and implementation science, which both concern ‘the challenges of translating intentions into desired changes’ ([1], p. 1). The focus is the large-scale investment in the Swedish National Quality Registries (NQRs), which are part of the Swedish quality management system described by the Organization for Economic Cooperation and Development (OECD) as having a depth and breadth that few other OECD countries can currently emulate [2]. Medical registries (also called quality or disease registries, clinical and medical databases, etc. [3],[4]) have been identified as a promising route for improved outcomes in health care and Sweden has been swift to adopt medical registries [5].

In 2014, 81 NQRs and 24 NQR candidates, as well as 8 regional competence centers, receive central funding in Sweden [6]. According to a national policy agreement, over 167 million Euros will be invested in the NQRs between 2012 and 2016. In addition to facilitating registry-based research, the policy agreement aims to increase the use of NQR data in efforts to improve health care [7], which we focus on in this study. The vision is that NQR data is used to follow up the processes and outcomes of clinics, hospitals, and county councils (local authorities responsible for financing, planning, and providing health care) [6]. Thus, NQRs may enable continuous quality improvement (QI) at the clinical level (where NQR data is used for identifying improvement areas, planning improvement efforts, and studying the result) and at the political and administrative levels (where NQR data is used to monitor performance against standards and locally set targets and goals). At the politico-administrative level, the use of NQRs has characteristics of both QI and audit [8].

It was, however, recently questioned whether the national policy agreement creates the right incentives for providers to enter correct data in the NQRs and to use the NQRs for QI [9]. The NQRs’ usefulness in research (generating generalizable knowledge) is rather well documented (e.g., [10]), whereas the understanding of how the NQRs contribute to quality improvement in the health service (focusing on internal processes that aim to improve the local situation) is in its early stages [11]. Currently, the evidence is fragmented as to how the use of national medical registries and the like lead to improved care. Effects are often poorly measured or studied without the possibility of controlling for effects of other simultaneous interventions [12],[13]. Reviewing the effectiveness on the quality of care, van der Veer et al. [14] concluded that trust in quality of the data was crucial for the effectiveness of feedback through medical registries. As important was the adding of components to the feedback initiative, for example, QI initiatives tailored by local teams, and not to forget, organizational factors (i.e., contextual factors).

**Figure 1:**
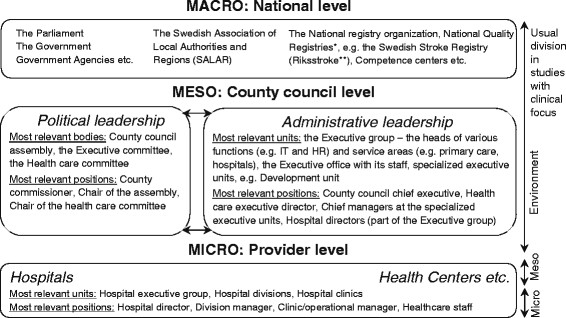
**Description of the levels within the Swedish health system.** *At the national level, each NQR is led by a national registry manager and a steering committee. A local registry manager is responsible for the registry at hospitals or primary care centres. **All 72 acute care hospitals that care for stroke patients are participating and the coverage is 91 per cent of all Swedish stroke patients (in total about 375,000 patients in the registry). Each year between 25,000 and 26,000 unique care episodes are included.

It is increasingly acknowledged that understanding success in implementation and QI requires the examination of context across all levels of the health care system, from the team or group level to the market/policy level [15],[16]. As Swedish health care is decentralized, the state’s intentions—for example, the use of NQRs for QI—are implemented by the 21 self-governing *county councils*. The county councils are led by democratically elected *politicians* and *administrators* (an umbrella term for, e.g., public officials, public servants, and public managers): which together form the *local politico-administrative* context for the service providers, hospitals, clinics, and staff. Most importantly, the local politico-administrative level (constituting the meso-level in the Swedish health care system, see Figure 1) decides on the county council budget and fees, establishes goals and guidelines for providers and staff, and commissions services from private and public providers. Monitoring and follow-ups are increasingly important tasks, in particular to secure the quality of care, for which the county councils are accountable to the public [17]. How the NQRs are integrated in the politico-administrative efforts to improve care quality has not yet been established. Generally, the use of medical registries by non-clinicians is not well researched, although this type of registries has become central in the political agenda in Europe and in other countries all over the world [18]. For example, research from the UK suggests that it is unclear whether medical registries are used for informing or evaluating services and policies at the provider level, and at national policy levels [3].

Thus, in the study, we examined implementation in the local politico-administrative context by interviewing key informants. The aim of the study was to investigate the perspectives of Swedish county council politicians and administrators on quality improvement based on national registry data. Primarily, we focused on their perspectives on politico-administrative use of NQRs in efforts to improve health care, but their perspectives also encompass hospital clinic’s use of NQR data for improving care. In an earlier study, we investigated the clinicians’ perspectives [11]. To investigate the politico-administrative context of the NQRs, we used the Consolidated Framework for Implementation Research (CFIR). The CFIR offers an overarching typology that may be used for evaluating implementation progress and to assess the implementation context [15]. In this study, we defined both context and quality improvement broadly. Quality improvement refers to any type of planned or continuous ‘actions for improving the processes and outcomes of health care’ [19] involving the NQRs, also including the continuous effort to secure high data quality—being pointed out as essential in previous research [14]. The politico-administrative context refers to the non-clinical conditions and activities that surround the implementation of NQRs and QI, which also ultimately creates the institutional/organizational conditions for the clinical use of NQRs for QI.

## Methods

### Sample and data collection

In Sweden, the self-governing county councils may organize their structure for provision and decision making as well as how they work with QI relatively freely. The implementation of the national agreement’s intent to increase the use of NQRs in QI is studied at the local politico-administrative level (the meso-level); see Figure 1. Significant for the politico-administrative level is that its representatives can make decisions applying to all providers in the county council. In this study, the macro-level (corresponding to the CFIR domain of outer setting) refers to overall national structures and institutions such as the national parliament and the national registry organizations. The micro-level refers to the provider level, comprising hospitals, clinics, health care staff, etc. Decisions on the clinical level apply only to its own operations.

Four county councils were included in the study (representing 23% of Sweden’s population). These were selected on the basis of being different in terms of size, population structure, hospital structure, political and management organization, and outcomes in the Swedish Stroke Registry (Riksstroke)—a sample created to allow for a subsequent comparison of actual NQR outcomes and contextual factors.

We used a *key informant* approach [20]—the chosen politicians and administrators being proxies for their associates within the organization. In each county council, 4 or 5 representatives were interviewed for a total of 17 individuals. These representatives occupied key positions, as presented in Table 1, and are together referred to as the politico-administrative leadership(s). To retain the informants’ anonymity, the respondents’ positions but not their locations are indicated when quotations are presented.

**Table 1:** **Description of the informants**

Position	Description of role	Respondents
County commissioner	Politician working full time governing the county council and responsible to the highest decision-making body, the assembly.	4
Health care executive director	The highest ranking administrator responsible for health care.	3
Chief manager of central development unit	Administrator who is the director of and ultimately responsible for a central development unit.	3
Co-worker at central development unit	Administrator working at a central development unit.	4
Hospital director	The highest ranking administrator of a hospital. All county councils do not have hospital directors.	3

The representatives were contacted by email or a printed letter and asked to participate. Five representatives declined, stating that they lacked the time to participate or that they had had their current position too short time to contribute with any relevant information. When participation was declined, substitutes were identified among those with a similar position in the county council. To ensure flexibility and thus increase the possibility for participation, the informants were interviewed via telephone (for between 30 and 50 min). The interviews were recorded and transcribed verbatim. Fourteen interviews were conducted by the first author, and the remaining three by a research assistant instructed by the first author, both using the same predefined semi-structured interview guide. Prior to the interview, the informants were informed about the purpose of the study and their right to withdraw from it at any time. The study was approved by the Research Ethics Board in Uppsala, Sweden (no. 2013/181), and informed consent was obtained before each interview.

The interview guide focused on the informants’ perspectives on how the county council works with NQRs and QI and who is responsible, how the different organizational levels work with NQRs and QI, how registry data and results are reported and shared between different levels in the organization, and what kind of supportive structures for working with NQR data and QI exists, as well as the current advantages and disadvantages of using NQRs in QI. More particular, the informants were asked about quality improvement related to the Riksstroke—the registry that targets the single somatic disease group that causes the highest number of hospital days in Swedish health care [21]. The purpose was to capture the politico-administrative views on a NQR that is well managed, long running, and affects a broad group of citizens and patients [22].

### CFIR—the analysis framework

The CFIR developed by Damschroder and colleagues [15] guided the analysis as a coding framework. The CFIR is a ‘meta-theoretical’ framework that synthesizes constructs from a range of theories about dissemination, innovation, organizational change, implementation, knowledge translation, and research uptake. It comprises five domains and 39 constructs (see Table 2). The five domains are 1) the characteristics of the intervention, 2) the outer setting, 3) the inner setting, 4) the characteristics of individuals, and 5) the process of implementation. Although the CFIR is often used for studying the implementation of well-defined clinical interventions, it has been described as applicable to a wide variety of situations and investigations across multiple contexts are encouraged to build a broader knowledge base [15].

**Table 2:** **Consolidated Framework for Implementation Research: domains and constructs and operationalized descriptions**

CFIR domains and domain descriptions	CFIR constructs and sub-constructs	Operationalized descriptions of the constructs and sub-constructs presented in the study
*Intervention characteristics:* characteristics of the intervention, which is often complex and multi-faceted, with many interacting components. Generally it has ‘core components’ and an ‘adaptable periphery’.	• Intervention source	*Evidence strength and quality:* validity and reliability, e.g., coverage and relevance of NQR variables
	• Evidence strength and quality	*Relative advantage:* not only the advantage of ‘the intervention versus alternative solutions’ but also advantages—and disadvantages—of the intervention, NQRs themselves
	• Relative advantage	*Design quality and packaging:* data input and data output (access) solutions
	• Adaptability	
	• Trialability	
	• Complexity	
	• Design quality and packaging	
	• Cost	
*Outer setting:* Generally, the outer setting includes the economic, political, and social context within which an organization resides.	• Patient needs and resources	*Cosmopolitanism:* networks and communications with actors external to the county council, i.e., with the NQR organizations, SALAR, public agencies, etc.
	• Cosmopolitanism	
	• Peer pressure	
	• External policy and incentives	
*Inner setting:* Generally, the inner setting includes features of structural, political, and cultural contexts through which the implementation process will proceed.	• Structural characteristics	*Structural characteristics:* the design of the politico-administrative organization (and the design of the clinical organization), e.g., the existence of quality improvement units
	• Networks and communications	*Networks and communications:* the presence of formal and informal communication regarding NQRs and care quality
	• Culture	*Tension for change*: current quality improvement efforts needs to be changed and based on NQRs
	• Implementation climate: *tension for change*, *compatibility*, *relative priority*, *organizational incentives and rewards*, *goals and feedback*, *learning climate*	*Relative priority:* Is the NQRs a prioritized data source when improving care?
	• Readiness for implementation: *leadership engagement*, *available resources*, *access to knowledge and information*	*Organizational incentives and rewards:* incentives and rewards intended to increase the quality of care through the use of NQRs
		*Goals and feedback:* Are there goals linked to NQR variables?
		*Leadership engagement*: commitment and involvement of politicians and administrators in the work related to NQRs and QI
		*Available* r*esources:* the level of resources available for work related to NQRs and QI
*Characteristics of individuals:* the individuals involved with the intervention and/or implementation process. Individuals have agency and are carriers of cultural, organizational, professional, and individual mindsets, norms, interests, etc.	• Knowledge and beliefs about the intervention	Not presented in the study
	• Self-efficacy	
	• Individual stage of change	
	• Individual identification with organization	
	• Other personal attributes	
*Process:* Implementation requires an active change process aimed to achieve individual and organizational level use of the intervention as designed. The implementation process may be an interrelated series of sub-processes that do not necessarily occur sequentially.	• Planning	Not presented in the study
	• Engaging: *opinion leaders*, *formally appointed internal implementation leaders*, *champions*, *external change agents*	
	• Executing	
	• Reflecting and evaluating	

Source: Damschroder et al. [15].

### Analysis procedure

Two researchers worked in parallel to analyze the interviews using content analysis [23]. To begin, each transcribed interview was coded for elements of the CFIR. Important passages in the interviews (meaning units) were coded into the relevant domains and constructs of the CFIR. All meaning units were then summarized into short codes that captured the essence of the statements. The short codes were later used when comparing the statements within the domains as well as between county councils. As a next step, the researchers met to discuss the interpretation of codes and to review coding discrepancies and then revised their individual codes based on a consensus regarding the meaning of each code. Consensus was reached by joint critical review and revisiting, if necessary, every single code in a quarter of the interviews. Some of the interviews contained about 100 codes.

In the coding phase, the full range of domains and constructs were used, although it is an option to work only with selected constructs [24],[15]. The final analysis, however, was constructed of three of the domains—the characteristics of the intervention and the inner and outer settings—into which the absolute majority of interview material was coded. As we did not focus on individual behavior change, the informants’ perceptions about the intervention were coded into ‘Characteristics of the intervention’ rather than into ‘Characteristics of individuals’ (see [24]). All constructs within the three domains were not touched upon by the respondents. Thus, similarly to Damschroder and Lowery [24], we relied on a ‘menu of constructs’ approach including only those that applied most directly to the present study.

Some adjustments of the placement of constructs were made. The ‘leadership engagement’ construct was broadened and also includes findings from the ‘engagement’ construct (originally in the ‘process’ domain). ‘Available resources’ also include ‘costs’ (in the ‘characteristics of intervention’ domain).

## Results

### Intervention characteristics

#### Evidence strength and quality

The politicians’ and administrators’ perception of *evidence strength and quality* was consistent among informants as well as between the county councils. However, it expressed a paradox. In general, the politico-administrative leaderships expressed great faith in NQRs and their role in QI, although they recognized potentially large problems with the *reliability* of the data. Problems often mentioned were that the staff (refers hereinafter to the health professionals at the hospital clinics) does not have enough time to register data, which causes information lags; the staff does not register the same kind of data; and errors occur when the staff transfers data from the electronic patient record (EPR) to the NQRs. Therefore, most of the informants mentioned that data needs to be analyzed carefully when planning improvement efforts based on NQR data, both at clinical and politico-administrative levels. Concerning data *validity*, the politicians and administrators expressed trust in the national NQR organizations’ judgement about the relevance of the included variables. However, in the light of the high costs of collecting NQR data, some questioned whether it is actually necessary to collect all the information currently collected.

#### Design quality and packaging

In terms of *design quality and packaging*, the politico-administrative leaderships stated, in a similar way, that the current data input and data output solutions constitute barriers to the broad assimilation of the intervention into the local health care organization. In their view, it was a major problem that data input is not automatized, for example, through the interlinking of EPRs and NQRs, especially as it exhausts the clinical staff. Data output and access to data were also major concerns. Most respondents mentioned that to initiate politico-administrative QI efforts, they need to attain output data in real time. Today, the NQR data is not instantly accessible to the politico-administrative leaderships. A health care executive director said, ‘One drawback is that too little NQR data is available online and accessible for those who are not involved in the actual data registration process. Increased accessibility and transparency would facilitate our analysis and our follow-ups and make our work more up to date’. One administrator working at a central politico-administrative development unit warned that politico-administrative leaderships risk acting on the wrong problem when they have to wait for the annual NQR reports.

#### Relative advantage

The politico-administrative leaderships described the *relative advantage* of using NQRs for QI in a consistent way. They compared the NQR data with the data in the EPRs and annual benchmark reports partly based on NQR data (*Quality and Efficiency in Swedish Health Care - Regional Comparisons* (*Öppna Jämförelser*)). They spoke of NQRs as an outstanding data source for QI which enables both internal and external benchmarking. Despite the NQRs’ advantages, most respondents mentioned that the politico-administrative leaderships instead of using NQRs actively work with QI based on the regional comparisons that are presented annually by the national authorities. Several of the respondents acknowledged that the regional comparisons can give a misleading picture, as the report is based on performance results that are up to 2 years old and that NQR data provides a more comprehensive and updated picture. Most notably, politicians preferred the regional comparisons to NQR data because they are easier to access, more readily available, and, in contrast to the specific NQRs, cover a range of different health care areas. Thus, in practice, the regional comparisons have a higher relative advantage than the NQRs.

### Inner setting

The inner setting, which includes ‘features of structural, political, and cultural contexts through which the implementation process will proceed’ ([15], p. 5), refers to the entire county council organization, i.e., the meso-level (politicians and administrators) and the micro-level (clinics). Our primary focus is the meso-level.

#### Structural characteristics

*Structural characteristics* concern both organizational structures and distribution of roles and responsibilities. From the politico-administrative leaderships’ descriptions, the structural characteristics were found to be both complex and irregular. In most county councils, several units within the formal politico-administrative organization as well as formal and informal networks were involved in work associated with NQRs and QI: creating a coordination challenge. Examples of politico-administrative units and networks were medical specialist councils, health care improvement centers, knowledge management teams, and regional registry centers. Yet, the respondents in all county councils could identify the existence of one central politico-administrative unit engaged in QI in their own county council. However, these units had a range of different assignments and NQRs were thus not their sole focus. Some of the respondents working in such units however remarked that they do not devote enough effort to analyzing data and engaging in QI.

The informants gave few examples of the formation of specialized politico-administrative structures to suit NQR-based improvement efforts better. One county council had integrated the local NQR managers of large registries in the politico-administrative organization by forming a permanent working group on NQR matters. Furthermore, two of the county councils had appointed a person at the politico-administrative level with overall responsibility for NQRs. One of the county councils lacking this cohesive function did not see this as a politico-administrative responsibility, but stressed that NQRs and subsequent QI efforts are a responsibility for each clinic. An organizational structure specific to improving stroke care through process management, also involving politico-administrative representatives, existed in three of the county councils. The administrators with insight into these processes acknowledged NQR data as the primary data source in this kind of process management.

There was a consensus among the politico-administrative representatives that the medical clinics are the core implementers, since the clinics produce the data and can directly use NQR data for QI. However, apart from the shared view on the clinics, a relatively mixed picture of responsibilities and roles emerged. Views on politicians’ responsibility and degree of involvement in QI and NQR work were the most fragmented. The most common opinion was that the role of politicians is to support quality improvement at the clinical level. However, the informants were vague when they talked about what political support means. Still, politicians considered themselves to be ultimately responsible for QI in the county councils, also when it is based on NQR data. Several politicians mentioned that they need to assume a more active role than they do today and also develop the politico-administrative use of NQR data, for instance, when they follow up whether the contracted providers deliver quality care.

#### Communication and networks

Communication of information on quality of care within the county council was complex and carried out via formal as well as informal channels. For example, politicians were informed about quality of care (e.g., NQR outcomes) in several ways: directly by the clinics, via development units/managers and through presentations to the county council executive committee or similar committees. Some of the politicians also looked through annual registry reports themselves. The launch of the regional comparisons creates most attention. When they are published, a manager at the central politico-administrative development unit normally gathers operations managers or talk to them separately, asking for their analysis of the outcomes and responses or plans for QI. How clinical operations managers progress after such meetings was not always clear to the responsible politicians and administrators. When asked how the operations managers act, if, for example, they talk to their staff after meeting politico-administrative representatives, a chief manager of a central politico-administrative development unit said, ‘That I cannot answer. I think they’re talking about the results, but I really do not know if they do it in any structured way’. When it comes to NQR results, the same approach was generally used, but NQR results could also be managed in less formalized ways; according to the politico-administrative leaderships, specialist councils and medical advisers have important roles as monitors of the clinics’ NQR results.

#### Implementation climate

A mixed picture of the *implementation climate* was conveyed by the politico-administrative leaderships. Importantly, there was a positive attitude toward the use of NQRs such as the Riksstroke and QI based on NQR data. Still, the *tension for change* was weak at the politico-administrative level as its representatives mainly relied on other types of performance data, e.g., the regional comparisons. Furthermore, several informants stated that cross county council efforts to develop the use of performance data to work out improvement strategies were based on the regional comparisons. Thus, at the politico-administrative level, the NQRs were given lower *relative priority* as a source of quality monitoring.

When it comes to *incentives and rewards*, the politico-administrative representatives described variation in the structure; two of the county councils had tied target-based compensations to NQR variables to improve outcomes. One example from Riksstroke was to reduce the door-to-needle time—that is, the interval between stroke patients’ arrival at the hospital and the start of thrombolytic treatment—from 62 to 40 min. Several informants also saw public reporting of NQR results as incentives to work with QI based on NQR data. As one health care executive director described it, ‘there is no one who wants to be worst’. All county councils had set *goals* for stroke care either in the overarching county council plan or in the clinics’ operational plans—goals which were partly possible to follow up via the Riksstroke variables.

#### Readiness for implementation

*Readiness for implementation* differs from *implementation climate* by its inclusion of tangible and immediate indicators of organizational commitment to implement an intervention [15].

*Leadership engagement* may be understood in different ways in relation to QI and NQR work. Sometimes, the informants defined engagement in NQRs as more of a moral support than a manifest engagement. One health care executive director said that the verbal support for NQR work that (s)he is communicating to the administrative staff is really important. Some respondents, however, also told of examples of more manifest leadership engagement in, for instance, the structure surrounding the stroke process in one of the county councils. In the ongoing process of improving stroke care, the politico-administrative leadership was represented in the project steering committee and also initiated the improvement project. None of the informants mentioned the existence of a committed and accountable leadership at all levels, i.e., at political, administrative, and clinical levels. A recurring statement was that the politico-administrative level was said to be very interested in NQRs and NQR results—an interest that rarely seemed to result in robust leadership engagement. Although the politico-administrative representatives sometimes participated in structures and activities built up around performance data and QI—such as target setting—this kind of leadership engagement was not necessarily perceived as support at the clinical level. For example, one of the politicians mentioned that health professionals such as physicians and registered nurses at the clinics sometimes disagree with the politicians concerning improvement areas and targets, and there is a scuffle between politics and clinics with regard to which health care performances should be measured. Informants from one county council claimed that the politico-administrative level, which had hitherto not been engaged in the improvement of care quality, was often not aware of ongoing QI efforts.

Regarding *available resources*, the politico-administrative representatives pointed out that the meso-level often lacks resources to analyze NQR-data, not least personnel resources. However, they primarily talked about the resources available at the clinical level. Several informants pointed out that it is costly to register and to use the NQR data. One chief manager of a politico-administrative development unit said that both politicians and administrators underestimate the resources needed to enter and use data from the NQRs. The informants pointed out that the clinics are offered no additional resources for undertakings associated with the NQRs, which are considered part of the clinics’ essential obligation to work with QI. The resources available are those of the clinics' regular budget, within which QI efforts are to be carried out, be they NQR-related or not. Some of the respondents mentioned that additional resources from the politico-administrative level can temporarily be deployed if a health care unit or process has particularly poor results. This happened in one of the studied county councils in which poor outcomes in stroke care some years ago prompted the politico-administrative level to initiate an improvement of the stroke care process.

Discussing resources available for QI, most informants considered the clinics’ resources for the NQR work and analysis of results data inadequate. Many of the informants argued that at least part of the financial resources in the national policy agreement should be directed to the county councils (instead of being handed to the national NQR organizations) and that the county councils today get very little back from the investment in NQRs.

### Outer setting

#### Cosmopolitanism

The county councils’ *cosmopolitanism*—that is, their networking with external organizations—related to NQRs and QI was described similarly by the politico-administrative leaderships. Overall, politicians, administrators, and professionals had a large number of contacts with the Swedish Association of Local Authorities and Regions (SALAR) that represents the county councils. Many informants had a positive picture of SALAR efforts. However, some of the administrators suggested that the SALAR does not focus on supporting the politico-administrative level in the process of improving care based on NQR data by, for example, supporting the internal building of supportive structures, but instead focuses on arranging clinical networking events for registry managers and similar activities.

The interviews highlighted that the politico-administrative leaderships do not cooperate or communicate with national NQR organizations such as the Riksstroke. Instead, the national organization of the Riksstroke communicates directly with the stroke clinics, and stroke clinics often turn directly to the Riksstroke for support in matters regarding data input, data quality, and so on. Also, administrators directly involved in the county council’s stroke process, such as process leaders and methods support staff, stated that they do not cooperate with the Riksstroke staff or board.

## Discussion

Using the CFIR, we have studied local politico-administrative representatives’ perspectives on quality improvement based on Swedish national registry data (with a few examples relating to the Swedish Stroke Registry, Riksstroke). We discuss the main observations from the three CFIR domains used in the study (intervention characteristics, inner setting, and outer setting) in the light of the national policy agreement and previous research on medical registries and QI.

Black and Tan [3] have suggested that in the UK, the underuse of national clinical databases at the policy level is due to a lack of awareness of such databases, an underestimation of the quality of the databases, and a lack of adequate funds to carry out analyses. In contrast, the Swedish interviews suggest that the politico-administrative leaderships are aware of the existence of the NQRs, but similarly, they have some doubts about the data quality and they point to a lack of resources available to analyze data. Furthermore, in contrast to Black and Tan’s comprehension that the data required for policy use is often readily available in the public arena, the lack of real-time access to NQR-data was perceived as a main barrier by the politico-administrative leaderships. In practice, lagging data access at the politico-administrative level (clinics can, however, access their own data in real time) makes it difficult for the politico-administrative leaderships to initiate, monitor, and support timely QI efforts. This reflects that the NQRs started as profession-led registries for epidemiological and medical research [25],[26]. Thus, the analysis of the intervention characteristics indicates that the NQRs need to be further adapted to be systematically used in politico-administrative efforts to improve care. As the regional comparisons (annual benchmark reports) more explicitly target the politico-administrative leaderships, these are currently preferred as a source for QI at the meso-level.

Little is known about how to improve care consistently across a variety of settings [16]. Integration across the organization’s boundaries [27] and effective communication across structural boundaries within the organization [28] have been pointed out as important for implementation and for improving care, conditions found to be inadequate in our study. The analysis of the inner setting shows that formal and informal units and networks dealing with NQR matters are not clearly linked together by efficient and distinct communication channels, which makes successful implementation less likely [28]. Also contributing to the complex structure is the county councils’ outer setting, where one substantial feature is the lack of communication and cooperation between the local politico-administrative leaderships and national NQR organizations such as the Riksstroke. The clinical stroke units communicate directly with Riksstroke, which in some cases, may be problematic as the NQR may have other opinions on how to develop registry work and quality of care compared to the local politico-administrative leaderships.

van der Veer et al. [14] mention the availability of resources and support by the management as important contextual factors in improving care by using medical registries. Neither at the politico-administrative level nor at the clinical level are the resources to analyze and use NQR data sufficient, negatively influencing the readiness for implementation. Furthermore, deficiencies in leadership engagement in relation to NQRs and QI at the politico-administrative level were described. The shortcomings of the politico-administrative leadership may be linked to the fact that politicians and administrators have dual roles in increasing the use of NQR data. Firstly, the politico-administrative level constitutes an important part of the implementation context for the clinics and thus has a responsibility to act in a way that supports the clinics in their use of NQR data when improving care. Today, the politico-administrative leaderships endorse the clinics’ participation in NQRs such as the Riksstroke, but do not require the clinics’ QI efforts to be based on NQR data. Secondly, the politico-administrative leaderships are also users of NQR data to control and monitor clinics and to create proper incentives for clinics to improve care. This use of NQR data is not yet assimilated in the county councils, although some of them have introduced monetary incentives linked to NQR performance.

At large, the interviews with the local politico-administrative leaderships indicate that the county councils’ support resources and structures are fragmented and not sufficient to analyze and act on NQR data as a tool for development and improvement [9],[29]. Thus, our findings suggest that the NQRs are not yet the institutional catalysts for efforts to improve outcomes over time, as suggested by Larson et al. [5]. For the advantages of the NQRs to be utilized and sustainable, the meso-level infrastructure and processes needs to be developed (see [29]).

One of the proposed advantages of the CFIR is that the framework can be used to build a knowledge base of findings across multiple contexts [15]. Usually, the CFIR is used to study more well-defined interventions such as a weight management program [24], not broad policy agreements implemented in the health service. As such policies are generally less specific (one of the challenges in policy implementation research is to define what is actually being implemented [1]), it may be difficult to use all the constructs and sub-constructs in the CFIR. The multiplicity in the policy agreement we studied may thus be one of the reasons why few statements were coded into the process domain (compare [30]), which deals with how implementation is planned, executed, and evaluated. To fully understand what the lack of process data means, more information is required. It may be that the CFIR does not capture the broader policy implementation process, but it may also be a reflection of the lack of an implementation strategy in the national policy agreement. Furthermore, studies using the CFIR usually focus on the clinical levels: the provider team or group level, hospital or clinical management level. In contrast, we focused on the non-clinical level which determines the clinics’ conditions. Often, the non-clinical tier is approached as the outer setting (i.e., the economic, political, and social context within which an organization resides [15]). However, we dealt with this level as part of the county councils’ inner setting as the politico-administrative level includes features of structural and political context through which the implementation process proceeds [15]. This approach creates many tiers within the inner setting, which may confuse the findings and conceal what actually works where and why—but reflects complexity in health service provision. Thus, the CFIR should acknowledge that there may be multiple tiers in the inner setting, some of which are non-clinical.

This study has both strengths and limitations. One of the strengths is that it tries out the use of the CFIR at the politico-administrative level, building the knowledge base of findings across multiple contexts, as suggested by Damschroder et al. [15]. One of the potential weaknesses is that the politico-administrative representatives did not always have the detailed knowledge we were asking for, for instance, regarding specific quality improvement efforts based on the NQRs and the use of the Riksstroke registry. In general, their views centered on the NQRs more broadly and sometimes approached the desired effects of using NQRs rather than actual experiences. As pointed out in another interview study using the CFIR, a qualitative approach might not reveal actual behavior [30]. However, the fact that the key informants did not always have the detailed knowledge is also important information. It indicates that the politico-administrative leaderships are not very involved in the implementation, which reduces the possibility of success and sustainability. Although we created a set of key informants that reflected several perspectives [20] on politico-administrative use of NQRs in improving care, it cannot be ruled out that another set of informants would have contributed with different views due to having different experiences or other personal or ideological approaches to how to govern the health services.

## Conclusions

The present study shows that to understand why and how the ‘desired changes’ come into being in complex systems, studies of implementation at the clinical level need to be complemented with studies of the clinics’ wider context, in this case, the politico-administrative level that regulates the institutional conditions within which the clinics operate. Thus, the Swedish experiences illustrate that a government-supported national system of well-funded, well-managed, and reputable national quality registries needs a supportive structure in the local health service in order for its data to be used for local quality improvement. Such conditions are not yet in place according to the interviewed politicians and administrators.

## Authors’ original submitted files for images

Below are the links to the authors’ original submitted files for images. Authors’ original file for figure 1

## Abbreviations

CFIR: Consolidated Framework for Implementation Research
EPR: Electronic patient record
NQR: National Quality Registry
OECD: Organization for Economic Cooperation and Development
QI: Quality improvement
SALAR: Swedish Association of Local Authorities and Regions
