# Supplementary figures and images for: Local politico-administrative perspectives on quality improvement based on national registry data in Sweden: a qualitative study using the Consolidated Framework for Implementation Research
Source: Implement Sci. 2014 Dec 28;9:189. doi: 10.1186/s13012-014-0189-6 (PMC4307376; doi:10.1186/s13012-014-0189-6)

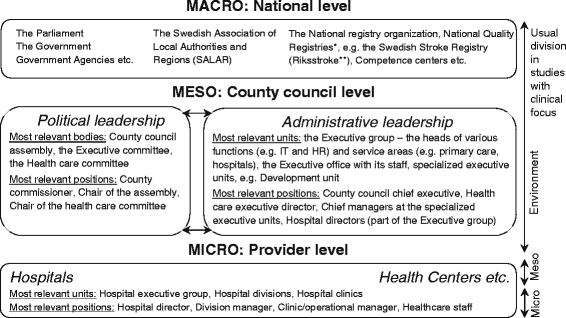

Supplement: Supplementary file 1 — Authors’ original file for figure 1 [file 13012_2014_189_MOESM1_ESM.gif]
